# Development and validation of Malaysia Medication Adherence Assessment Tool (MyMAAT) for diabetic patients

**DOI:** 10.1371/journal.pone.0241909

**Published:** 2020-11-06

**Authors:** Ernieda Hatah, Nadiah Rahim, Mohd Makmor-Bakry, Noraida Mohamed Shah, Noraini Mohamad, Mahadir Ahmad, Nor Hasni Haron, Choe Sze Hwe, Angeline Tan Meng Wah, Fahmi Hassan, Shakirin Shaik Rahmat, Sarah Ann Robert, Noraidatulakma Abdullah

**Affiliations:** 1 Faculty of Pharmacy, Universiti Kebangsaan Malaysia, Jalan Raja Muda Abdul Aziz, Kuala Lumpur, Malaysia; 2 Pharmaceutical Care Branch, Pharmacy Practice and Development Division, Pharmaceutical Services Programme, Ministry of Health Malaysia, Jalan Universiti, Petaling Jaya, Selangor, Malaysia; 3 Health Psychology Programme, Faculty of Health Sciences, Universiti Kebangsaan Malaysia, Jalan Raja Muda Abdul Aziz, Kuala Lumpur, Malaysia; 4 Pharmacy Department, Universiti Kebangsaan Malaysia Medical Center, Jalan Ya’acob Latiff, Bandar Tun Razak, Cheras, Kuala Lumpur, Malaysia; 5 UKM Medical Molecular Biology Institute (UMBI), Universiti Kebangsaan Malaysia, Jalan Ya’acob Latiff, Bandar Tun Razak, Cheras, Kuala Lumpur, Malaysia; Witten/Herdecke University, GERMANY

## Abstract

Medication non-adherence remains a significant barrier in achieving better health outcomes for patients with chronic diseases. Previous self-reported medication adherence tools were not developed in the context of the Malaysia population. The most commonly used tool, MMAS-8, is no longer economical because it requires a license and currently every form used is charged. Hence, there is a need to develop and validate a new medication adherence tool. The Malaysia Medication Adherence Assessment Tool (MyMAAT) was developed by a multidisciplinary team with expertise in medication adherence and health literacy. The face and content validities of the MyMAAT was established by a panel of experts. A total of 495 patients with type 2 diabetes were recruited from the Ministry of Health facilities consisting of five hospitals and five primary health clinics. A test-retest was conducted on 42 of the patients one week following their first data collection. Exploratory factor analysis was performed to evaluate the validity of the MyMAAT. The final item for MyMAAT was compared with SEAMS, HbA_1c_%, Medication Possession ratio (MPR) score, and pharmacist’s subjective assessment for its hypothesis testing validity. The MyMAAT-12 achieved acceptable internal consistency (Cronbach’s alpha = 0.910) and stable reliability as the test-retest score showed good to excellent correlation (Spearman’s rho = 0.96, p = 0.001). The MyMAAT has significant moderate association with SEAMS (Spearman’s rho = 0.44, p = < 0.001) and significant relationship with HbA1c (< 8% and ≥ 8%) (χ^2^(1) = 13.4, p < 0.001), MPR (χ^2^(1) = 13.6, p < 0.001) and pharmacist’s subjective assessment categories (χ^2^(1) = 31, p < 0.001). The sensitivity of MyMAAT-12, tested against HbA1c% was 72.9% while its specificity was 43%. This study demonstrates that the MyMAAT-12 together with other methods of assessment may make a better screening tool to identify patients who were non-adherence to their medications.

## Introduction

Adherence to medication refers to whether patients take their medications as prescribed by the doctors and continue taking the prescribed medications [1]. Poor adherence to medications can lead to negative outcomes on patients’ health such as suboptimal clinical benefit, and hospitalization, and may also lead to death [2]. The problem of non-adherence to medication may arise due to many reasons such as patient-related factors (e.g. having negative beliefs and/or perceptions on medications), condition-related factors, socioeconomic-related factors, and therapy-related factors (e.g. complexity of treatment regime and pill burden) [3]. An example of non-adherence to medication is when patients have a misconception about their medication, they may intentionally decide to stop taking it [4,5]. Other reasons for medication non-adherence include carelessness, forgetfulness and poor health literacy [6].

The prevalence of poor medication adherence is reported to be high worldwide. It is estimated that in developed countries, 50% of patients with chronic diseases do not have good adherence to their medication treatment plan [2]. Patients have a higher risk of medication non-adherence if they are suffering from chronic diseases and/or receiving multiple types of medications [7]. It has been estimated that 30% to 50% of patients prescribed with medications for different types of chronic diseases in the United States are non-adherent to their medication therapies. Medication non-adherence is also reported to be high in developing countries such as Gambia and China with non-adherence rates of 73% and 54%, respectively [8].

In Malaysia, despite considerable subsidization of the cost of medications in public health care settings, the prevalence of poor adherence is still high. A study by Aziz et al. among patients with chronic diseases receiving care at public hospitals and clinics found an adherence rate of only 50% [9]. Earlier studies among patients with diabetes and hypertension receiving treatment in public hospitals and clinics also reported low adherence rates of 53% and 53.4%, respectively [10,11]. A study by Hassali et al. (2012) found that the returned unused subsidized medications in a public hospital in Malaysia were high, with an average cost of RM42.35 per patient. The postulated cost of returned unused subsidized medications across Malaysia is estimated to exceed a few million dollars per year [12]. The results of the study, however, is perceived as an underreporting since it only included patients who returned the unused medications at a hospital counter or received home visits by pharmacists. The wastage of medications caused by poor adherence will cause a huge economic burden for the government, both short- and long-term, by reducing the government’s resources and worsening patients’ disease conditions.

Medication non-adherence may occur at any stage of the medication-taking process. Some patients may exhibit medication non-adherence at the beginning of their treatment. However, throughout the treatment duration, patients may also stop taking the treatment for many reasons [6]. Thus, an assessment of patients’ adherence to medication must be conducted throughout the process when patients take their medications. In clinical practice, healthcare practitioners such as doctors and pharmacists are usually required to emphasize medication adherence to their patients. This is important to assess the patients’ reasons for stopping medications and to help to resolve those issues so that medication effectiveness and patients’ health outcomes can be optimized. In addition, it is important to identify medication non-adherence to avoid possible treatment effects from being underrated [13]. Therefore, it is important to have a reliable, valid, and cost-effective tool to measure patients’ adherence to their medications [14]. The unavailability of a standard medication adherence assessment tool may cause health care practitioners to use their own judgment about patients’ adherence status which may be varied, inconsistent, and difficult to be replicated, even by the same practitioner.

Many tools and methods of assessing patients’ medication adherence have been developed in the past decade. In general, adherence can be assessed using subjective and objective methods. The objective assessments include the measurement of clinical outcomes such as target disease biomarkers, pharmacy records, dose count, and drug concentrations as well as using electronic monitoring of medication administration such as Medication Event Monitoring System (MEMS) [13]. Examples of subjective measurements for medication adherence include reports from a patient’s physician or family, patient interviews, and self-report adherence scales [13]. An ideal medication adherence measure should have a low cost, be user-friendly, feasible, highly reliable, flexible, and practical. However, this is difficult to achieve due to the well-known limitations of each measurement tool [15,16].

Self-report questionnaires are preferable in a busy, resource-limited clinical setting and population that has moderate to high literacy. The systematic review done by Nguyen et al. had identified 43 validated English versions of self-report adherence scales [13]. However, many of these tools were developed overseas, which may not be suitable for Malaysian patients. This limits its usefulness in the Malaysian setting. Moreover, the Malaysian Medication Adherence Scale (MALMAS), which closely resembles that of the widely used Morisky Medication Adherence Assessment Scale (MMAS), is also bound to MMAS’s license for usage [17]. Starting in 2015, researchers or practitioners were charged US$1 per form of English MMAS and US$1.50 for an MMAS prepared in another language [18]. The tool, therefore, is no longer cost-effective to be used in Malaysia’s clinical settings in which assessment may include patients who attend health services such as Medication Therapy Adherence Clinics, Home Medication Review, or normal counselling and a follow-up at hospital and clinics. For these reasons, this study aims to develop and validate a medication adherence assessment tool to be used in the Malaysian health care practice.

## Methods

### Background and conceptual framework of the instrument

The Malaysia Medication Assessment Adherence Tool (MyMAAT) was developed through a literature review of previously developed and validated medication adherence assessment tools by Nguyen et al., and by exploring local expert opinions on Malaysian patients’ medication adherence behaviour [9]. This tool was also developed based on reported health psychology theories of behavioural changes that focus on medication adherence [19]. Based on reported psychological theory, common predictors for medication adherence were identified and included in the questionnaire. Consistent predictors of adherence that was selected were self-efficacy such as the sociocognitive, self-regulation, and social support, underpinned by the general control beliefs theory. Previous measurement tool for multiple sclerosis reported self-efficacy to correctly classify up to 98.8% of adherence cases at six-month follow-up [19]. Other important predictors such as patients’ perceived barriers or concern (93% consistency), adverse effects or general harm, benefits of the medications or necessity (correctly classified 74% cases), severity (correctly classified 50% of cases), susceptibility (correctly classified 86% of cases) and overuse of medication explained by the Health Belief Model-sociocognitive theory and belief about medicines-self-regulation theory were also included in the questionnaire [19,20].

The commonly asked questions in previously reported adherence assessment tools were divided into three categories following its specific constructs. The identified constructs were developed based on the categorization made by Nguyen et al. in their systematic review study which are: i) specific medication-taking behaviours: e.g. dose taken, dose frequency, dose administration, and prescription refills; (ii) barriers to adherence: e.g. forgetfulness, treatment complexity, and side effects; and/or (iii) beliefs associated with adherence: e.g. perceived necessity of medicines and concerns about medicines [13]. Combining the commonly asked questions with important predictors of medication adherence, five constructs were identified to be the basic for the development of MyMAAT. These were: 1) patients’ medication-taking behaviour, 2) perceived utility of the medications–benefits, costs, and efficacy; 3) perceived barriers to medication adherence; 4) perceived self-efficacy and social support; and 5) perceived severity and susceptibility of diabetes. Other factors that may predict patients’ medication adherence as reported by the local qualitative study by Aziz et al. (2018) and local expert opinion that is ‘having many medications stock at home’ was also included in the initial instrument [21]. The initial 21 items of MyMAAT and its preliminary constructs are presented in Table 1.

**Table 1:** The initial 21 items of MyMAAT and its preliminary constructs.

Construct	Item number	Items
**Medication taking behaviour**	1	*In the past month*, *I frequently failed to take my medication in accordance with the doctor’s instruction*.
	2	*In the past month*, *I reduced my medication intake when I felt better*.
	3	*In the past month*, *I took my medication alternately*.
	4	*I was often late for / missed the appointment date to get the supplies of my follow-up medication at the pharmacy counter*.
Others*	5	*I have an excess supply of the prescribed medication at home*.
**Perceived utility (Benefits, costs and efficacy)**	6	*In my opinion*, *the medication prescribed to me did not work well enough to improve my health condition*
	7	*I feel that my medication is not effective*.
	8	*I feel that my overall health condition will be better without medication*.
	9	*I did not fully comply with the prescriptions because I felt it was unnecessary/ insignificant*
**Perceived barriers**	10	*In the past month*, *I frequently failed to remember to take my medication*.
	11	*I often feel uninterested in taking my medication*.
	12	*I regularly take less medication than prescribed for fear of the side effects to my body*.
	13	*In the last month*, *I frequently forgot to bring along my medication with me when I went out*.
	14	*I fear that taking medication continuously will result in over reliance on medication*.
**Sociocognitive theory (self-efficacy and social support)**	15	*Compliance to a medication intake routine/schedule is a challenge for me*.
	16	*I will miss/not take my medication if no one reminds me to do so*.
	17	*I am uncertain about my daily medication doses*.
	18	*I am unable to manage my medication intake properly*.
	19	*Without support or help from the loved ones*, *I lack motivation to take my medication as prescribed by the doctor*.
**Perceived severity and susceptibility**	20	*My illness is not serious*.
	21	*In my point of view*, *my illness will not deteriorate although I miss/did not take my medication*.

*Based on local expert opinion.

### Content, linguistic, and face validity

All reported adherence assessment questionnaire located were evaluated and categorized into related constructs by NR, EH and MMB. The questions and its related constructs were then presented to the expert teams that consisted of academicians, a clinical psychologist, a statistician, the Pharmaceutical Care Section of the Pharmacy Practice and Development Division Team from the Pharmaceutical Services Programme responsible in planning, developing, and coordinating pharmaceutical care services such as medication counselling, Medication Therapy Adherence Clinic (MTAC) and clinical pharmacy services in Ministry of Health (MOH) facilities, and 10 pharmacist practitioners who provide the diabetes MTAC services in government hospitals and primary health clinics. The expert team was asked to review the constructs and its questions and provide their opinion and agreement on questions that should be included or added into the adherence assessment tool.

Based on the expert team’s opinion, the initial instrument consisted of 21 questions from five constructs that were assembled and prepared in Malay. The forward translation to English was done by a professional English translator and an expert team member who happened to be a clinical psychologist and known to use English in his daily communication. The language validation was checked by two members of the expert team who chose the best translation. Another two independent practitioners, who were known to use both Malay and English in their daily communication helped to do the backward translation to the Malay language. Any disagreement was resolved through consensus. The item-content validity index (I-CVI) and scale-validity index (S-CVI) were further assessed by four independent pharmacist practitioners and one academician who was also a medical practitioner. All were independent reviewers and not included in the expert team. Reviewers were asked to rate the content relevance using four ordinal Likert-scale items which were 1 = not relevant, 2 = somewhat relevant, 3 = quite relevant, and 4 = very relevant. The score was then categorized as ‘content invalid’ which consisted of ratings 1 and 2, whereas ratings of 3 and 4 are considered to be ‘content valid’. The I-CVI was calculated by counting the number of expert reviewers giving a score of 3 or 4 and dividing it by the total number of experts. S-CVI was the proportion of items on the scale that achieved a relevance scale of 3 or 4 by all expert reviewers. The cut off point for the I-CVI score was set at 1.00 which indicates complete agreement on ‘content valid’ (rating 3 and 4) for tool with five judges or fewer [22] and an S-CVI of > 0.8 was considered an acceptable content validation value [23].

A five-point response scale range was used to score the instrument. This Likert-scale response allowed respondents to express how much they agreed or disagreed with a particular statement [24]. Patients were required to state the level of their agreement of strongly disagree, disagree, neutral, agree, and strongly agree on statements relating to their medication adherence behaviour. The potential score for the 21-item scale ranged from 21 to 105, with each ‘strongly disagree’ to ‘strongly agree’ response receiving between five marks and one mark, respectively. Higher scores indicated a better medication adherence rate. A pilot test was conducted for the first 20 patients in each centre (n = 10) who participated in the study. Pharmacists conducted the content and face validity to patients in the pilot study by asking them whether the individual MyMAAT item was easily understood, phrased appropriately, whether or not any specific items need to be rephrased for improvement, and if there was any difficulty in using the self-administered questionnaire (e.g. time required to complete the survey). Feedback from patients and pharmacists’ obtained from the pilot study were used to refine the instrument. Patients took approximately 5 to 10 minutes to complete the questionnaires in the pilot study.

### Criteria for participation and the data collection process

The initial MyMAAT was tested in 10 MOH facilities consists of five hospitals and five clinics from Perak, Selangor, Federal Territory of Kuala Lumpur and Putrajaya, Negeri Sembilan, and Johor states. The facilities were selected based on the availability of the pharmacy electronic system that was able to identify patients’ refill records. Using convenient sampling, patients with the following criteria were invited to participate in the study: (i) adult patients aged ≥ 18 years old, (ii) diagnosed with diabetes mellitus, (iii) prescribed with medications for diabetes, and (iv) currently are using the same diabetes regime for at least three months. Patients were excluded from the study if they were: (i) unable to give informed consent; (ii) unable to understand Malay or English language; (iii) caregiver-dependent; (iv) cognitively impaired such as with Alzheimer’s disease and/or dementia; (v) critically ill such, as patients at the final stage of cancer or HIV; or (vi) patients without a recent HbA1c% reading of less than six months. Prior to the data collection, patients were given an explanation about the study and were asked to sign the informed consent if they agreed to participate and allow the use of their medical records for the data collection. The following patients’ demographic and data were gathered during the study: i) age, gender, race, diagnosis, comorbidities, education level, and socioeconomic status; ii) two latest prescription dispensing and refill dates for Medication Possession Rate (MPR) calculation; and iii) the patients’ latest HbA1c% reading. All patients’ data were fully anonymized before the research team had accessed to it. Patients were asked to complete the MyMAAT initial instrument by themselves based on their behaviour when taking their anti-diabetic medication. Patients were informed and showed their anti-diabetic medications to avoid confusion with other medications and were reminded that they should answer the question by reflecting on their medication taking in the past month. In addition, pharmacists who conducted the survey were asked to do subjective assessments on patients’ adherence to medication. They could ask further questions to patients after the patients had completed the questionnaire for their assessment. Patients were categorized as adherent if they were presumed to take at least 80% of their medications; they were classified as moderately or poorly adherent by the pharmacists if they were suspected to take less than 80% of their medications. Patients were invited again to complete the survey after a week for retest, which is commonly done in questionnaire design to determine the test-retest reliability.

### Sample size calculation

Using the 20:1 subject to item ratio calculation for exploratory factor analysis suggested by Costello (2005), a minimum sample size of 420 patients was required for this study [25]. The sample size for a test-retest to determine the level of agreement for the adherence score of the same patients at two different periods was determined based on intraclass correlation coefficient (ICC) of 0.4, alpha of 0.05, power of 80% [26]. The required sample size was 36. An additional of 20% drop out rate was included in case patients failed to attend the follow-up session. Hence the number of sample size required was 43.

### Data analysis

All data were analysed using SPSS version 22.0. Descriptive analysis for respondents’ demographic and characteristics used number, percentage and mean with standard deviation where necessary. The exploratory factor analysis (EFA) was conducted to ensure the smallest number of factors that best represented the items [25]. Oblique rotation with the Direct Oblimin method was used because there were potential correlations between items measuring the same construct. Data were considered suitable for EFA analysis if the item correlation matrix showed > 0.30 and the Kaiser-Meyer-Olkin coefficient was > 0.70 [27,28]. Using parallel analysis with the Monte Carlo PCA, the number of factors were determined if the actual eigenvalue from EFA was higher than its criterion value through parallel analysis [29]. Item factor loadings and significance of factor loadings were evaluated to confirm the unidimensionality of the measure. Minimum acceptable factor loading was defined as 0.40 or higher, with 0.50 and above indicating strong loading; and p-value of 0.05 indicated a significant factor loading for an item [30]. Factors were considered weak if generally have less than three items and fewer than three items had a factor loading of ≥ 0.50.

The internal consistency of the tool was tested using Cronbach’s alpha. In the current study, Cronbach’s alpha of ≥ 0.70 was used as the cut-off point for good internal consistency. The inter-item correlation cutoff point of > 0.8 and < 0.2 were used to remove items that were too similar or did not correlate well with other items, respectively. Items that did not correlate well with the tool’s total score were also removed from the questionnaire. Only items with an item-total correlation of ≥ 0.3 were included in the questionnaire. A test-retest reliability was used to assess the stability of the measurement over time. The test-retest analysis was measured using the Intraclass Correlation Coefficient (ICC) of absolute agreement based on a two-way mixed model and interpreted following Rosner: ICC <0.40 = poor agreement, ICC 0.40–0.75 = fair to good agreement and ICC ≥ 0.75 = excellent agreement) [31].

Hypothesis testing with convergent validity of the revised MyMAAT was subsequently evaluated in terms of its association with Self-Efficacy for Appropriate Medication Use Scale (SEAMS) [32] and HbA1c% using Spearman’ correlation test. The score for MyMAAT was calculated as follows. Individual items were scored using the following criteria: five marks for ‘strongly disagree’, four marks for ‘disagree’, three marks for ‘neutral’, two marks for ‘agree’, and one mark for ‘strongly agree’. The MyMAAT score was the total of the marks for the individual items. Using the score that will provide the best sensitivity and specificity results, the revised MyMAAT was then grouped into two groups: (1) good adherence and (2) moderate and poor adherence. The validity of the group or known groups validity was then assessed against HbA_1c_ less tight categories of ≥ 8% and < 8% as stated in the Malaysia Diabetes Clinical Practice Guideline (2016) [33], Medication Possession Ratio (MPR) categories (≥ 80% and < 80%), and a pharmacist’s subjective assessment of the patient’s medication adherence categories (adherence and non-adherence) using Chi square (χ2) tests. A p-value of < 0.05 was considered as statistically significant. Sensitivity and specificity as well as the positive and negative predictive values (precision rate) of the MyMAAT score were performed with HbA_1c_%, MPR and pharmacist’s subjective assessment categories [34].

### Ethics approval

The study received ethics approval from the Medical Research & Ethics Committee, Ministry of Health Malaysia (NMRR-18-1329-42071).

## Results

### Patient characteristics

A total of 495 patients were recruited in the study. The patients’ demographic and characteristics are presented in Table 2.

**Table 2:** Respondents’ demographic and characteristic (n = 495).

Variables	n (%)	Mean ± SD^§^/Median (IQR)^≠^
**Age (years)**		57.2 (10. 8)^**§**^
**Gender**		
Male	266 (53.7)	
Female	229 (46.3)	
**Ethnicity**		
Malay	301 (60.8)	
Chinese	125 (25.3)	
Indian	68 (13.7)	
Others	1 (0.2)	
**Highest Education Level**		
No formal education	33 (6.7)	
Primary school	90 (18.2)	
Secondary school	248 (50.1)	
Tertiary school	75 (15.2)	
Undergraduate degree	30 (6.1)	
Postgraduate	15 (3.0)	
Others	4 (0.8)	
**Job Status**		
Employed	197 (39.7)	
Unemployed	98 (19.8)	
Housewife	75 (15.1)	
Pensioner	104 (21)	
Students	2 (0.4)	
Others	17 (3.4)	
**Monthly Income**		
No income	190 (38.1)	
< RM1000	56 (11.3)	
RM1000—RM2000	93 (18.8)	
RM2000 –RM3000	61 (12.3)	
RM3000 –RM5000	61 (12.3)	
≥RM5000	27 (5.4)	
**Insulin Users**		
Yes	165 (33.3)	
No	330 (66.7)	
**Num. of comorbidity**		2 (IQR 2–3)^≠^ (Range 1–5)
**Num. of medications**		5 (IQR 4–6)^≠^ (Range 1–12)
**HbA**_**1c**_ **(mmol/dL)**		7.96 (1.89)

### Content validity

All items in MyMAAT-21 had item-content validity index (I-CVI) and scale-validity index (S-CVI) of 1.00. The structure obtained a Kaiser-Meyer-Olkin coefficient of 0.92 with a χ^2^(21) = 5604.1, < 0.001. These data indicated that it was possible to conduct the factor analysis.

### Psychometric properties of MyMAAT

The items of the MyMAAT-21 were subjected to exploratory factor analysis (EFA), which aimed to examine the factor structure of the tool. Using Oblique rotation with Direct Oblimin analysis, a total of eight items were removed from the tool (Table 3). Item 13 which dealt with the perceived barriers and self-efficacy related to medication adherence of ‘frequently forgot to bring along medication when went out’ was removed because it did not meet the minimum acceptable factor loading of ≥ 0.40. Another three weak factors, which consisted of seven items (items 6, 7, 8, 11, 14, 20 and 21), were removed as the factor had fewer than three items or fewer than three items had factor loading of ≥ 0.50. These were items 6, 7, and 8 which dealt with the perceived utilities, such as benefits and efficacy of the medication in relation to adherence; items 11 and 14, which dealt with perceived barriers to medication adherence as ‘often feel uninterested to take medication’ and ‘fear that medication adherence may result in over reliance to medication’; and items 20 and 21, which dealt with perceived disease severity and susceptibility. The final principal components of the EFA consisted of a two-factor solution with a cumulative percentage of variance of 61.76% with 12 final items. The 12 final items of MyMAAT are available in S1 Table. Item 15, which is ‘compliance to a medication intake routine/schedule is a challenge for me’ was excluded because the factor loading reduced to < 0.40. The factor structure obtained is summarized in Table 4. Items 1, 2, 3, 4, 5, 9, 10, and 12 were loaded into factor 1 and items 16, 17, 18, and 19 were loaded into factor 2. These factors were felt to represent two clear dimensions of practice of specific medication-taking behaviour (factor 1) and reasons for medication non-adherence related to sociocognitive theory of self-efficacy and social support (factor 2). Items included were found to be the same even when the structure was forced into one-structure solution with reduced cumulative percentage of variance to 52.06%.

**Table 3:** Factor analysis for original scale (Direct Oblimin Rotated Component Matrix).

Items		Factor 1 Rotated Component Loading	Factor 2 Rotated Component Loading	Factor 3 Rotated Component Loading	Factor 4 Rotated Component Loading	Factor 5 Rotated Component Loading
Eigenvalue		9.06	1.54	1.20	1.11	1.01
% of variance explained		43.15	7.31	5.75	5.28	4.79
2	*In the past month*, *I reduced my medication intake when I felt better*.	0.69				
4	*I was often late for / missed the appointment date to get the supplies of my follow-up medication at the pharmacy counter*.	0.66				
3	*In the past month*, *I took my medication alternately*.	0.65				
10	*In the past month*, *I frequently failed to remember to take my medication*.	0.58				
5	*I have an excess supply of the prescribed medication at home*.	0.57				
1	*In the past month*, *I frequently failed to take my medication in accordance with the doctor’s instruction*.	0.57				
9	*I did not fully comply with the prescriptions because I felt it was unnecessary/ insignificant*	0.51				
12	*I regularly take less medication than prescribed for fear of the side effects to my body*.	0.49				
13	*In the last month*, *I frequently forgot to bring along my medication with me when I went out*.	< 0.40				
7	*I feel that my medication is not effective*.		0.85			
6	*In my opinion*, *the medication prescribed to me did not work well enough to improve my health condition*		0.52			
8	*I feel that my overall health condition will be better without medication*.		0.49			
18	*I am unable to manage my medication intake properly*.			-0.86		
16	*I will miss/not take my medication if no one reminds me to do so*.			-0.84		
19	*Without support or help from the loved ones*, *I lack motivation to take my medication as prescribed by the doctor*.			-0.71		
17	*I am uncertain about my daily medication doses*.			-0.57		
15	*Compliance to a medication intake routine/schedule is a challenge for me*.			-0.45		
14	*I fear that by taking medication continuously will result on over reliance on medication*.				0.69	
11	*I often feel uninterested in taking my medication*.				0.47	
21	*In my point of view*, *my illness will not deteriorate although I miss/did not take my medication*.					0.83
20	*My illness is not serious*.					0.48

**Note**: Item 13 were removed as factor loading < 0.40; Item 7, 6 and 5 were removed as less than 3 items had factor loading of > 0.50; Factor 4 (item 14 and 11) and Factor 5 (item 21 and 20) were removed due to weak factors as it contained < 3 items.

**Table 4:** Factor analysis of reduced items (2 factor).

Items		Factor 1 Rotated Component Loading	Factor 2 Rotated Component Loading
Eigenvalue		6.25	1.16
% of variance explained		52.06	9.69
Cumulative %		61.76	
1	*In the past month*, *I frequently failed to take my medication in accordance with the doctor’s instruction*.	0.56	
2	*In the past month*, *I reduced my medication intake when I felt better*.	0.75	
3	*In the past month*, *I took my medication alternately*.	0.73	
4	*I was often late for / missed the appointment date to get the supplies of my follow-up medication at the pharmacy counter*.	0.73	
5	*I have an excess supply of the prescribed medication at home*.	0.61	
9	*I did not fully comply with the prescriptions because I felt it was unnecessary /insignificant*	0.61	
10	*In the past month*, *I frequently failed to remember to take my medication*.	0.56	
12	*I regularly take less medication than prescribed for fear of the side effects to my body*.	0.60	
16	*I will miss/not take my medication if no one reminds me to do so*.		- 0.86
17	*I am uncertain about my daily medication doses*.		- 0.61
18	*I am unable to manage my medication intake properly*.		- 0.93
19	*Without support or help from the loved ones*, *I lack motivation to take my medication as prescribed by the doctor*.		- 0.72

**Note**: Item 15 “Compliance to a medication intake routine/schedule is a challenge for me” was removed as factor loading < 0.40.

### Reliability

Cronbach’s alpha coefficient with the reduced items, MyMAAT-12, was 0.91, which shows excellent internal consistency. Cronbach’s alpha would not have increased with the deletion of any items (Table 5). Item-total correlation coefficients revealed moderate to strong correlations of all 12 items to the total scale, ranging from 0.52 to 0.72. Inter-item correlation coefficients revealed moderate to strong correlations of the 12 items, ranging from 0.26 to 0.73, indicating that all items were within the recommended range of 0.2–0.8 and that no items were exceedingly redundant or did not correlate well with other items. Based on responses from 42 patients who completed the follow-up interview, the ICC for test-retest reliability of the 12-item scale was 0.97 (95% CI 0.93 to 0.98), indicating high stability.

**Table 5:** Reliability analysis of MyMAAT-12.

Item Number	Corrected item total correlations	Cronbach’s alpha if item deleted	Test-retest reliability Wilcoxon signed rank test (p-value)
1	0.659	0.902	0.083
2	0.612	0.904	**0.025**
3	0.710	0.900	**0.046**
4	0.565	0.907	**0.025**
5	0.522	0.910	**0.002**
6	0.721	0.900	0.083
7	0.691	0.900	0.059
8	0.687	0.900	**0.008**
9	0.674	0.902	**0.014**
10	0.662	0.902	0.180
11	0.721	0.900	0.059
12	0.619	0.904	**0.025**

### Hypothesis testing validity

The final scale of the MyMAAT-12 score showed a significant moderate association with medication adherence assessed by Self-Efficacy for Appropriate Medication Use Scale (SEAMS) (Spearman’s ρ = 0.44, p-value <0.001), providing evidence for the convergent-related validity of the MyMAAT-12 scale. In addition, the MyMAAT-12 score also had significant weak inverse association with patients’ HbA_1c_% level (Spearman’s ρ = -0.20, p-value <0.001). For known groups validity, a score between 12 and 54, and 55 and 60 was used to classify patients into adherence and non-adherence categories, respectively. About 63.8% (n = 316) patients had a score of < 54, which was considered as non-adherence, and another 36.2% (n = 179) had a score ≥ 54, which was categorized as adherence by MyMAAT-12. The MyMAAT-12 categories also able to show a significant different between patients who were adherent and non-adherent to their medications according to HbA_1c_% (< 8% and ≥ 8%) (χ^2^(1) = 13.4, p <0 .001), MPR (χ^2^(1) = 13.6, p <0 .001) and pharmacist’s subjective assessment categories (χ^2^(1) = 31, p < 0.001) (Table 6).

**Table 6:** MyMAAT-12 categories’ known groups validity.

**MyMAAT-12 score**	**HbA**_**1c**_**% (n, %)**		**χ**^**2**^ **(df)**	p-value*
	**≤ 8%**	**>8%**		
Moderate and poor adherence (score < 54)	160 (50.6)	156 (49.4)	13.4 (1)	< 0.001
High adherence (score ≥ 54)	121 (67.6)	58 (32.4)		
**MyMAAT-12 score**	**Medication Possession Ratio (n, %)**		**χ**^**2**^ **(df)**	p-value*
	**Non-adherence < 80%**	**Adherence ≥ 80%**		
Moderate and poor adherence (score < 54)	62 (19.6)	254 (80.4)	13.6 (1)	< 0.001
High adherence (score ≥ 54)	13 (7.3)	166 (92.7)		
**MyMAAT-12 score**	**Pharmacist’s subjective assessment**		**χ**^**2**^ **(df)**	p-value*
	**Non-adherence < 80%**	**Adherence ≥ 80%**		
Moderate and poor adherence (score < 54)	129 (49.4)	132 (50.6)	31 (1)	< 0.001
High adherence (score ≥ 54)	37 (22.4)	128 (77.6)		

*Pearson Chi-square test.

### Sensitivity and specificity

As shown in Table 7, the MyMAAT-12 sensitivity using HbA_1c_%, MPR and pharmacist’s subjective assessment categories were 72.9%, 82.7% and 77.7%, respectively. While the MyMAAT-12 specificity using HbA_1c_%, MPR and pharmacist’s subjective assessment were 43%, 39.5% and 49.2%, respectively. The positive and negative predictive values were 49.4% and 67.6% with HbA_1c_% categories, 19.6% and 92% with MPR categories, and 49.4% and 77.6% with pharmacist’s subjective assessment categories.

**Table 7:** Sensitivity and specificity test for MyMAAT-12.

**MyMAAT classification (n = 495)**	**Poor control HbA**_**1c**_ **> 8%**	**Good control HbA**_**1c**_ **≤ 8%**	**Positive and negative predictive value**
**Moderate & poor adherence (score < 54) n (%)**	156 (49.4%) [TP]	160 (50.6%) [FP]	**Positive PV** TP / (TP + FP) × 100% = 49.4%
**Good adherence (score ≥ 54) n (%)**	58 (32.4) [FN]	121 (67.6) [TN]	**Negative PV** TN / (TN + FN) × 100% = 67.6%
**Sensitivity and specificity**	**Sensitivity** TP / (TP + FN)×100% = 72.9%		**Specificity** TN / (TN + FP)×100% = 43%
**MyMAAT classification (n = 495)**	**Poor adherence MPR < 80%**	**Good adherence MPR > 80%**	**Positive and negative predictive value**
**Moderate & poor adherence (score < 54) n (%)**	62 (19.6) [TP]	254 (80.4)[FP]	**Positive PV** TP / (TP + FP) × 100% = 19.6%
**Good adherence (score ≥ 54) n (%)**	13 (7.3) [FN]	166 (92.7) [TN]	**Negative PV** TN / (TN + FN) × 100% = 92%
**Sensitivity and specificity**	**Sensitivity** TP / (TP + FN)×100% = 82.7%		**Specificity** TN / (TN + FP)×100% = 39.5%
**MyMAAT classification (n = 495)**	**Pharmacist’s subjective assessment (Non-adherence)**	**Pharmacist’s subjective assessment (Adherence)**	**Positive and negative predictive value**
**Moderate & poor adherence (score < 54) n (%)**	129 (49.4) [TP]	132 (50.6) [FP]	**Positive PV** TP / (TP + FP) × 100% = 49.4%
**Good adherence (score ≥ 54) n (%)**	37 (22.4) [FN]	128 (77.6) [TN]	**Negative PV** TN / (TN + FN) × 100% = 77.6%
**Sensitivity and specificity**	**Sensitivity** TP / (TP + FN)×100% = 77.7%		**Specificity** TN / (TN + FP)×100% = 49.2%

PV, Predictive value; TP, True positive; TN, True negative; FP, False positive; FN, False negative; MPR, Medication possession ratio.

## Discussion

The current study has developed the MyMAAT-12 that can be used to assess medication adherence among patients with diabetes. The overall scale of MyMAAT-12 as determined by psychometric testing can be considered as reliable and valid with weak to moderate significant correlation with HbA1c% and the self-reported SEAMS. Factor analysis of the MyMAAT-12 revealed two dimensions of medication self-efficacy which are i) practices of specific medication-taking behaviour that relate with non-adherence and ii) reasons for medication non-adherence. The findings of this study are consistent with the previous reported factors of medication adherence. These are the *self-efficacy*, *self-regulation*, *and social support theory and also the perceived necessity of medication referring to concern related to adverse effects* or *general harm and benefits of the medications* [19]. Explained by *self-regulation theory*, which is the regulated behaviour of the pursuit of one’s own goal [35], it was found that the patients’ motivation to be compliant to medication, perceived controlled over illness, responsibility to comply, and perceived support received for medication compliance are significant predictors for medication adherence [36]. Identifying the underpinned reasons for medication non-adherence around these two dimensions may help to identify patients’ problems in medication taking and facilitate interventions that tailor to patient needs.

The MyMAAT-12 was found to have excellent internal consistency with Cronbach’s alpha coefficient of 0.91 [37]. Removal of any items would not increase the results to more than 0.91. Hence, retaining the 12 items of MyMAAT was reasonable. In addition, the re-test analysis with the 12 items shows that it had stable reliability. The tool was also shown to have valid convergent and known groups validity against other adherence measurement tools. It shows a significant moderate association with SEAMS scores, which was previously tested against MMAS [13]. However, unlike SEAMS which did not have significant association with HbA_1c_%, the current tool had a significant weak inverse association with the biomarker for the glycemic control. The result was found to be similar with the findings from the MALMAS study conducted in Malaysia, which has a development in close resemblance to MMAS-8 items [17]. In MALMAS study, the tool was reported to have weak inverse correlation with HbA_1c_% with Spearman’s rho of -0.212, p = 0.014. The weak significant association between the MyMAAT score and HbA_1c_% could be due to the reason that HbA1c% test may not truly represent medication adherence alone as it is also confined to other cofounding factors such as dietary intake and exercise.

The weak significant correlation with HbAc% resulted in the decision to categorize the scores into two groups of adherence and nonadherence using the cutoff score of 54. With the score cut-off point, the MyMAAT categories showed there were significant differences between HbA_1c_%, MPR and pharmacist’s subjective assessment categories between patients who were adherent and non-adherent to their medications. Patients who were classified as non-adherent by MyMAAT-12 were found to have significant higher mean of HbA_1c_% and lower mean for MPR and pharmacist’s subjective assessment than those who were classified as adherent. This indicated that MyMAAT-12 has good convergent and known groups validity with both objective and subjective assessments of medication adherence. Unlike other tools, MyMAAT-12 was tested not only against HbA1c% but also on three other measurement tools that use objective and subjective assessment approaches. This was the strength for MyMAAT-12 as usually a combination of more than one tool is needed to confirm medication adherence [38].

In the current study, MyMAAT-12 has the sensitivity of between 72.9% and 82.7%, and specificity of 39.5% and 49.2% when tested against HbA_1c_%, MPR and pharmacist’s subjective assessment. This means the ability of MyMAAT-12 to correctly identify those patients who are non-adherence or true positive, using the categories set by the tests are higher than its ability to correctly identify those patients who are adherence or true negative to their medication [34]. A tool can be considered as an accurate instrument if a high number of true positive and true negatives were obtained, when compared to false positives and false negatives. MyMAAT-12 was found to have lower sensitivity than previously developed medication adherence assessment tool in Malaysia known as MALMAS (88.9%) and comparable sensitivity with MMAS-8 validated for Malaysian version (77.6%) [17,39]. In term of specificity, MyMAAT-12 shows higher specificity than MALMAS (29.6%) and comparable specificity with MMAS-8 Malaysian version (45.4%). When measured against the referent standard, the tool has lower PPV percentage of between 19.6% and 49.4% than its NPV which is between 67.6% and 92%. This shows that MyMAAT-12 has lower probability in detecting true non-adherence among positively tested patients than its probability to detect true adherence among negatively tested patients [34]. The lower PPV than NPV of MyMAAT-12 was found to be in similar pattern with MALMAS and MMAS-8 Malaysian version. The PPV and NPV of MALMAS were reported to be 31.7% and 87.9% [17]. While the PPV and NPV for MMAS-8 Malaysian version were 46.84% and 76.56%, respectively [39]. In view of the results, it is perceived that MyMAAT-12 has the probability to include more patients of true positive (true non-adherence) and false positive (false nonadherence) than excluding those with false negative (false adherence), which is important not to be overlooked. In addition, since the PPV and NPV are dependent on the population being tested and are influenced by the prevalence of the problem [34], it is belief that if MyMAAT-12 is tested among patients with high suspicion for medication non-adherence, for example those with HbA_1c_% of more than 8%, the PPV will be increased. Because of the above, the tool is perceived to be useful to be use with other assessment methods to assist practitioners to investigate patients’ medication adherence status. In the case of the medication adherence support clinic in Malaysia or known as Diabetes Medication Therapy Assessment Clinic (DMTAC), MyMAAT can be used to further support the selection criteria for the service following HbA_1c_% of more than 8% [40].

The limitations of the study include that MyMAAT-12 was developed mainly using indirect sources of previously validated tools which were developed for overseas population. Nevertheless, the current study had ensured that the contents were relevant to the local population by testing it among practicing practitioners and patients. It is also well noted that majority of the content reviewers were pharmacists or academician in the pharmacy field, thus may limit the usefulness of this tool to other health profession. However, the tool is developed as self-administrated in which can be learn easily by other healthcare practitioners. In addition, the current tool was validated only in Malay and English languages. Thus, only patients that can read and understand Malay or English were included in the study. Nevertheless, although Malaysia is a multiracial nation consisting of Malay, Chinese, Indian and other ethnicities who may use other languages such as Mandarin and Tamil, the Malay and English languages are compulsory syllabi in the education curriculum in Malaysia in which the majority of population may be able to read and understand at least one of these languages. Moreover, the adherence assessment was done only for anti-diabetic medications and it did not cover adherence assessment for patients’ other medications. Since it is not uncommon that diabetic patients had other comorbidity that may require the use of other medications, generalizing the adherence status of this tool as an overall adherence may not be appropriate and need to be further tested. The fair sensitivity and low specificity result of the tool reflects that it is sensitive to detect non-adherence but has lack of ability to correctly predict adherence to diabetes medication.

## Conclusion

MyMAAT-12 together with other assessment methods, for example the patients’ HbA_1c_% levels, may make a better screening tool to identify patients who were non-adherence to their medications. The usefulness of the tool to facilitate the correct interventions as it includes identification of patients’ potential reasons for medication non-adherence, however, needs to be further explored.

## Supporting information

S1 Table The 12 items MyMAAT. (DOCX)
