# Supplementary material for: Development and validation of Malaysia Medication Adherence Assessment Tool (MyMAAT) for diabetic patients
Source: PLoS One. 2020 Nov 6;15(11):e0241909. doi: 10.1371/journal.pone.0241909 (PMC7647074; doi:10.1371/journal.pone.0241909)
Supplement: S1 Table — (DOCX) [file pone.0241909.s001.docx]

**Table S1. The 12 items Malaysia Medication Adherence Assessment Tool (MyMAAT)**

*Perception on Patient’s Adherence Towards Antidiabetic Medications. This survey will ask your current practice related to medicine taking in the past month. *Please tick ( ∕ ) in the appropriate boxes.*

| **No** | **Item** | **Score** | | | | |
| --- | --- | --- | --- | --- | --- | --- |
|  |  | Strongly disagree | Disagree | Neutral | Agree | Strongly agree |
| 1. | In the past month, I frequently failed to take my medication in accordance with the doctor’s instruction. |  |  |  |  |  |
| 2. | In the past month, I reduced my medication intake when I felt better. |  |  |  |  |  |
| 3. | In the past month, I took my medication alternately. |  |  |  |  |  |
| 4. | I was often late for / missed the appointment date to get the supplies of my follow-up medication at the pharmacy counter. |  |  |  |  |  |
| 5. | I have an excess supply of the prescribed medication at home. |  |  |  |  |  |
| 6. | I did not fully comply with the prescriptions because I felt it was unnecessary/ insignificant. |  |  |  |  |  |
| 7. | In the past month, I frequently failed to remember to take my medication. |  |  |  |  |  |
| 8. | I regularly take less medication than prescribed for fear of the side effects to my body. |  |  |  |  |  |
| 9. | I will miss/not take my medication if no one reminds me to do so. |  |  |  |  |  |
| 10. | I am uncertain about my daily medication doses. |  |  |  |  |  |
| 11. | I am unable to manage my medication intake properly. |  |  |  |  |  |
| 12. | Without support or help from the loved ones, I lack motivation to take my medication as prescribed by the doctor. |  |  |  |  |  |
